# Comparison of Human and Animal Surveillance Data for H5N1 Influenza A in Egypt 2006–2011

**DOI:** 10.1371/journal.pone.0043851

**Published:** 2012-09-27

**Authors:** Peter M. Rabinowitz, Deron Galusha, Sally Vegso, Jennifer Michalove, Seppo Rinne, Matthew Scotch, Michael Kane

**Affiliations:** 1 Yale School of Public Health, Yale University, New Haven, Connecticut, United States of America; 2 Yale Occupational and Environmental Medicine Program, Yale University, New Haven, Connecticut, United States of America; 3 Yale Center for Analytic Sciences, Yale University, New Haven, Connecticut, United States of America; 4 Department of Biomedical Informatics, Arizona State University, Phoenix, Arizona, United States of America; 5 Department of Internal Medicine, Yale University, New Haven, Connecticut, United States of America; University of Iowa, United States of America

## Abstract

**Background:**

The majority of emerging infectious diseases are zoonotic (transmissible between animals and humans) in origin, and therefore integrated surveillance of disease events in humans and animals has been recommended to support effective global response to disease emergence. While in the past decade there has been extensive global surveillance for highly pathogenic avian influenza (HPAI) infection in both animals and humans, there have been few attempts to compare these data streams and evaluate the utility of such integration.

**Methodology:**

We compared reports of bird outbreaks of HPAI H5N1 in Egypt for 2006–2011 compiled by the World Organisation for Animal Health (OIE) and the UN Food and Agriculture Organization (FAO) EMPRESi reporting system with confirmed human H5N1 cases reported to the World Health Organization (WHO) for Egypt during the same time period.

**Principal Findings:**

Both human cases and bird outbreaks showed a cyclic pattern for the country as a whole, and there was a statistically significant temporal correlation between the data streams. At the governorate level, the first outbreak in birds in a season usually but not always preceded the first human case, and the time lag between events varied widely, suggesting regional differences in zoonotic risk and/or surveillance effectiveness. In a multivariate risk model, lower temperature, lower urbanization, higher poultry density, and the recent occurrence of a bird outbreak were associated with increased risk of a human case of HPAI in the same governorate, although the positive predictive value of a bird outbreak was low.

**Conclusions:**

Integrating data streams of surveillance for human and animal cases of zoonotic disease holds promise for better prediction of disease risk and identification of environmental and regional factors that can affect risk. Such efforts can also point out gaps in human and animal surveillance systems and generate hypotheses regarding disease transmission.

## Introduction

The majority of emerging infectious diseases in recent decades are zoonotic (transmissible between animals and humans) in origin [1], a fact underscored by the recent global spread of novel H1N1 influenza A. As part of an effective global response to such disease emergence, there has been calls for integrated surveillance of zoonotic disease events in human and animal populations, although to date, there have been few examples of such data integration [2].

Highly pathogenic avian influenza (HPAI) H5N1 represents both an epizootic of enormous scope and a significant pandemic threat to human health. Since the onset of the current epizootic in 1996, extensive surveillance efforts in both animal and human populations have taken place on a global scale. While at present avian influenza remains predominantly an animal disease, with sporadic zoonotic transmission to humans and apparently limited human-to-human transmission, there is ongoing risk for the emergence of strains with increased transmissibility. It is therefore a public health priority to reduce human risk for avian influenza infection by controlling the infection at its source (animal populations), and also taking steps to reduce the possibility of animal-human (zoonotic) transmission. Avian influenza accordingly provides an opportunity to test the utility of integrating human and animal disease surveillance data streams.

**Figure 1:**
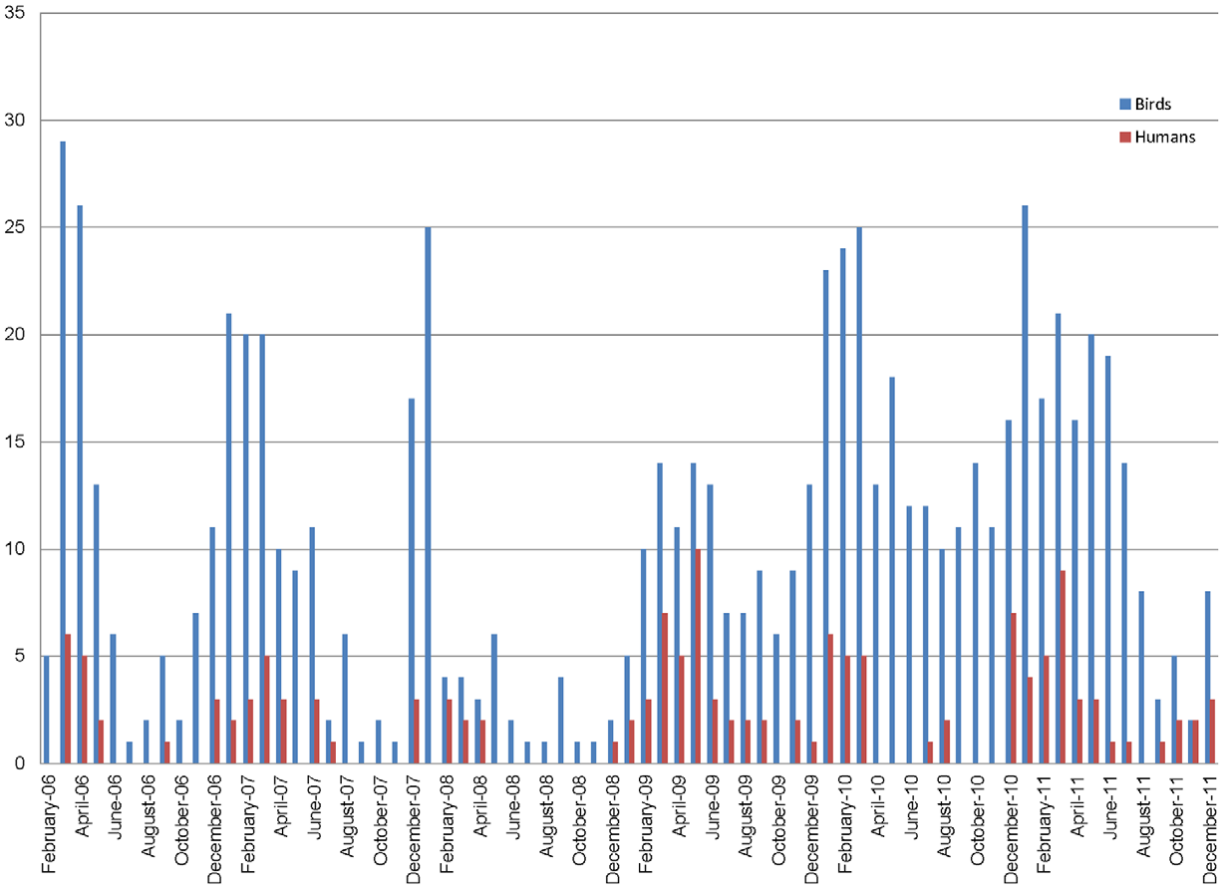
Epidemic curve for bird outbreaks and human cases of H5N1 in Egypt 2006–2011.

Currently, disease surveillance for cases of HPAI infection is carried out separately for humans and animals. Ministries of health in different countries report confirmed human cases of avian influenza to the World Health Organization (WHO), which lists these reports on the public WHO avian influenza website [3]. Meanwhile, national ministries of agriculture report outbreaks of avian influenza in birds to the World Organisation for Animal Health (OIE), which also makes public these disease reports. There is currently no official integration of these surveillance data streams.

**Figure 2:**
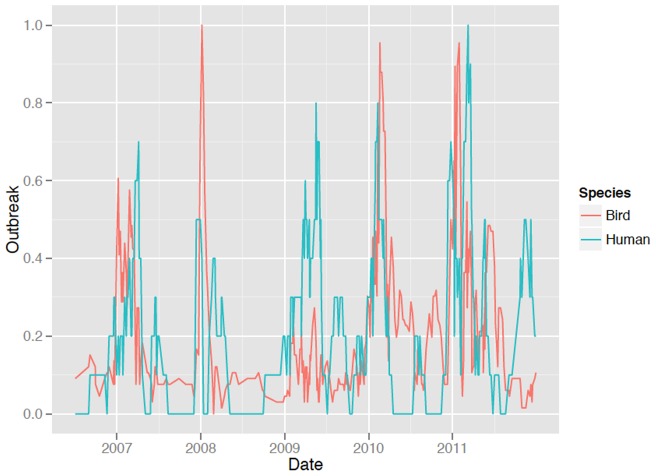
Normalized curves for human cases and bird outbreaks of H5N1 in Egypt.

To date, comparisons of human and animal HPAI surveillance data have been largely descriptive, such as displaying maps or tables showing both human and animal cases [4]. Such descriptive mapping reveals that there is a good deal of spatial overlap between human and animal disease occurrence. However, to date there has been little analysis of statistical relationships between human and animal surveillance data streams for HPAI, including the identification of risk factors for linkage between animal and human risk, or the creation of predictive models for forecasting and early intervention to prevent further human cases. Such integrated approaches to surveillance could improve prevention and control efforts for both human and animal health.

**Figure 3:**
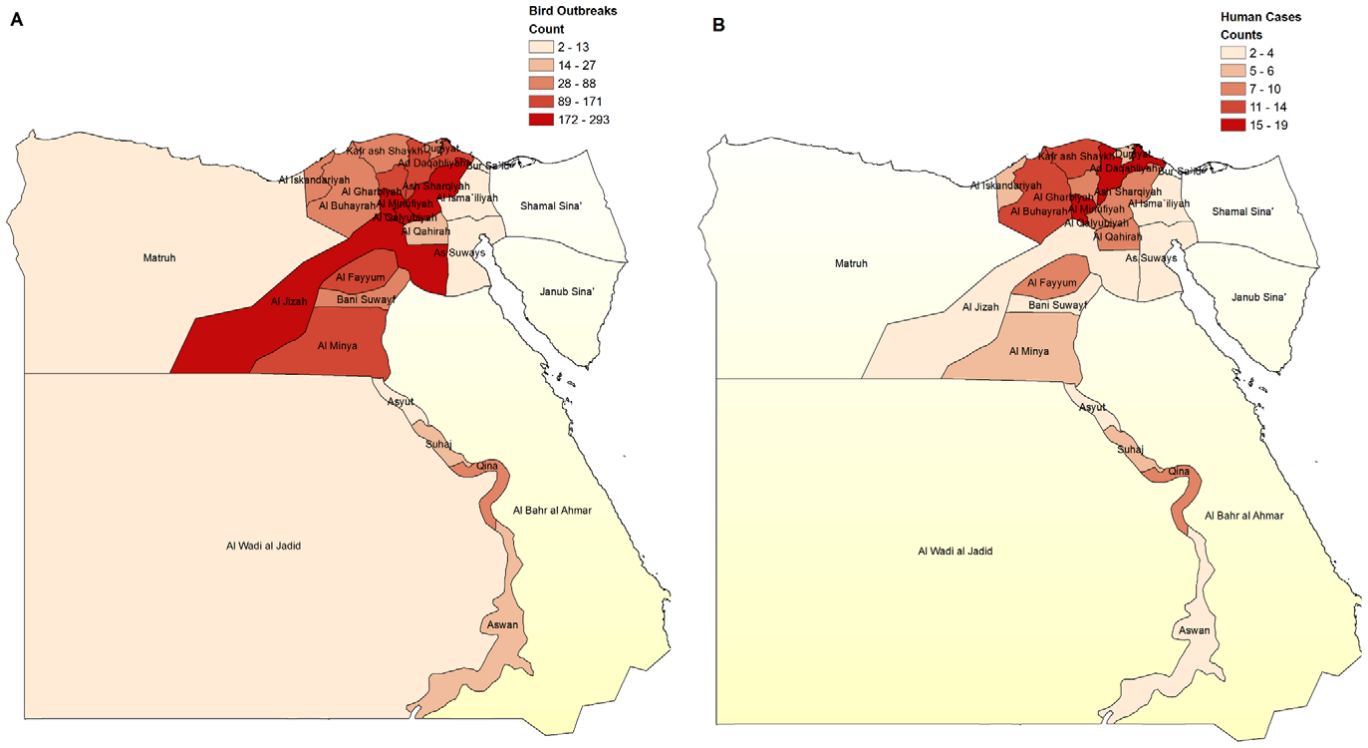
A. Bird outbreaks of H5N1 by governorate 2006–2011. B. Human cases of H5N1 by governorate 2006–2011.

**Figure 4:**
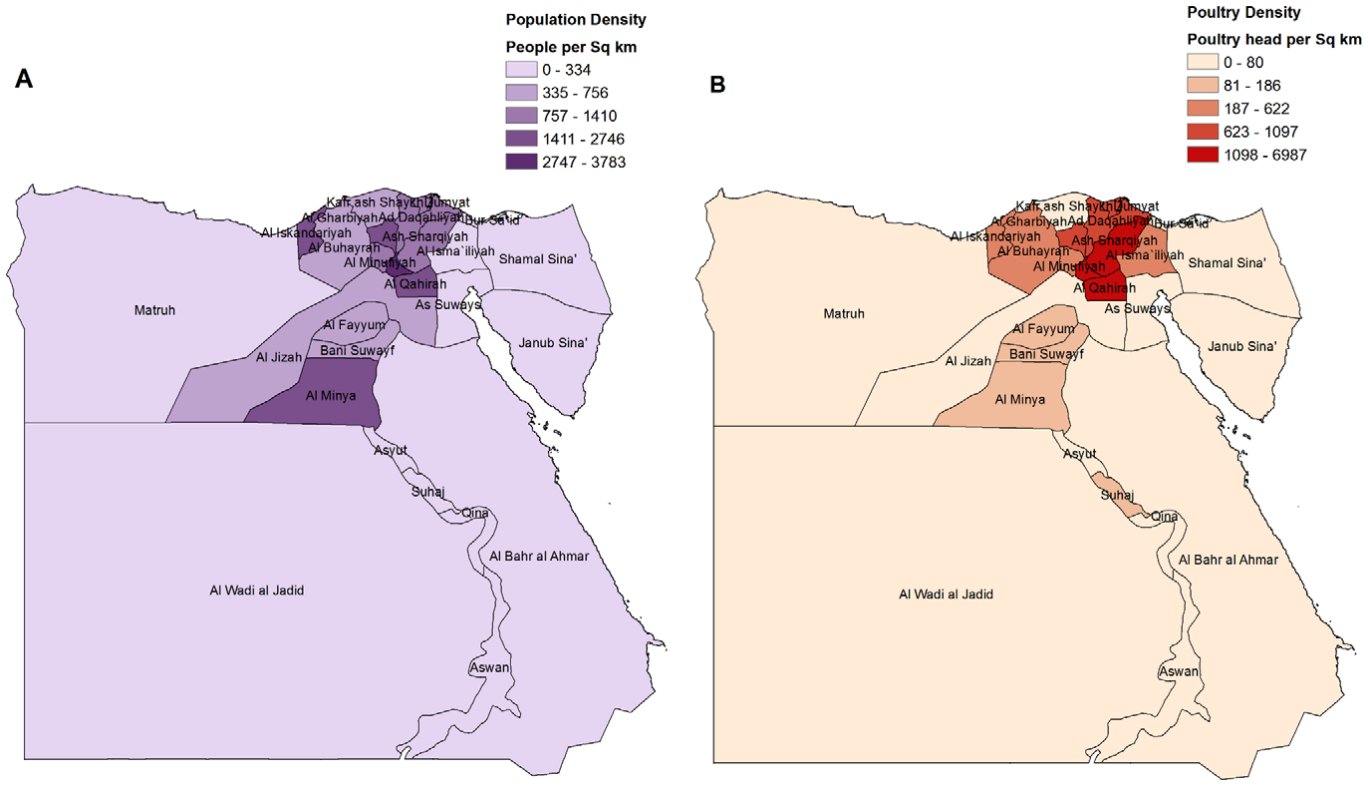
A. Population density map (people per square km), Egypt. B. Poultry density map (poultry head per sq km), Egypt.

Egypt is one of the countries most affected by HPAI in both animal and human populations. In 2006, at the same time that the virus was appearing in other African countries [5] there were widespread outbreaks in Egyptian commercial and domestic poultry flocks caused by a strain of influenza A H5N1 of Eurasian origin [6], possibly introduced into the country by wild birds [7], [8]. Shortly following the onset of these poultry outbreaks, the first human cases appeared in the country. As of June 2012, Egypt had reported 168 human cases of HPAI with 60 fatalities, making it the second most affected country in terms of human cases, after Indonesia [3]. Previous reports have noted that human cases tend to occur more in the winter months, and in the Nile Delta region, with the majority of cases appearing in individuals less than 18 years of age [9]. The ongoing occurrence of cases of H5N1 infection in both poultry and humans in Egypt places this region at risk of being a secondary epicenter for further spread of the disease [10]. While separate studies have reported on the epidemiology of avian influenza in Egypt in birds (source) and in humans [9], no studies to date have directly compared the two surveillance data streams.

**Table 1:** Number of days between first bird outbreak and human case for each season (9/1–8/31).

Governorate	9/2005–8/2006	9/2006–8/2007	9/2007–8/2008	9/2008–8/2009	9/2009–8/2010	9/2010–8/2011
Al Bahr al Ahmer						
Al Buhayrah			91	50	−96	−54
Ad Daqahliyah		145	115	−25	112	83
Al Fayyum	86	144	175	152	163	187
Al Gharbiyah	73	−4		210	82	128
Al Iskandariyah				36	−142	95
Al Jizah						88
Al Minufiyah	84		55	14	141	134
Al Minya	127	202	171	150	13	
Al Qahirah		46		63	−21	56
Al Qalyubiyah	63	206		10	94	262
Al Wadi al Jadid						
Al Ismàiliyah						
As Suways				−28		54
Ash Sharqiyah		164	223	96	118	58
Aswan		169				
Asyut				−58	125	
Bani Suwayf		84	111		60	
Bur Sàid						
Dumyat		294	114	−3	30	−30
Kafr ash Shaykh	80			11	45	130
Luxor						
Matruh						
Qina		68		−1		63
Suhaj	60	142		−3		
**Average**	**82**	**138**	**132**	**42**	**52**	**90**

We therefore performed an analysis of the relationship between reported surveillance cases of H5N1 highly pathogenic avian influenza in birds and humans in Egypt from 2006–2010. The objective of the study was to determine whether reports of bird outbreaks could be used to predict human infection risk, and to characterize the temporal relationships between the data streams.

**Table 2:** Predictors of human H5N1 case occurrence in a governorate.

	Bivariate				Multivariate			
Risk Factor	RR	95% CI		p-value	RR	95% CI		p-value
Mean temperature last 30 days	0.94	0.92	0.95	<.0001	0.94	0.92	0.96	<.0001
Mean humidity last 30 days	1.01	1.00	1.03	0.054				
Urbanization of Governorate	0.99	0.98	0.99	0.0002	0.99	0.98	1.00	0.0046
Poultry Density	1.45	1.19	1.76	0.0002	1.26	1.03	1.54	0.0218
GDP of Governorate	0.89	0.82	0.97	0.0056				
Ducks								
Outbreak with ducks in last 14-days	2.59	1.83	3.66	<.0001				
Outbreak with ducks in last 21-days	2.45	1.75	3.41	<.0001				
Chickens								
Outbreak with chickens in last 14-days	2.64	1.91	3.65	<.0001				
Outbreak with chickens in last 21-days	2.30	1.67	3.18	<.0001				
Ducks and Chickens								
Outbreak with ducks and chickens in last 14-days	2.48	1.70	3.61	<.0001				
Outbreak with ducks and chickens in last 21-days	2.36	1.66	3.35	<.0001				
Ducks or Chickens								
Outbreak with ducks or chickens in last 14-days	2.64	1.91	3.65	<.0001	1.91	1.37	2.67	0.0001
Outbreak with ducks or chickens in last 21-days	2.30	1.66	3.17	<.0001				

## Materials and Methods

We created and analyzed an integrated data set containing information from human and animal surveillance data streams as well as demographic and other risk information available at the governorate level for Egypt.

**Table 3:** Positive Predictive Value (PPV) for bird outbreaks predicting the occurrence of a human case of H5N1 HPAI in the same governorate within the next 14 days.

Time Period	# Bird Outbreaks	# with Human Case within 14 days	Positive Predictive Value
9/1/2005–8/31/2006	616	85	13.8
9/1/2006–8/31/2007	270	14	5.2
9/1/2007–8/31/2008	166	5	3.0
9/1/2008–8/31/2009	134	21	15.7
9/1/2009–8/31/2010	398	61	15.3
9/1/2010–8/31/2011	453	70	15.5
**Total**	**2037**	**256**	**12.6**

**Table 4:** Sensitivity for bird outbreaks predicting the occurrence of a human case of H5N1 HPAI in the same governorate within the next 14 days.

Time Period	# Human Cases	# of Bird Outbreaks within 14 days prior	Sensitivity
9/1/2005–8/31/2006	14	12	85.7
9/1/2006–8/31/2007	24	8	33.3
9/1/2007–8/31/2008	12	4	33.3
9/1/2008–8/31/2009	33	10	30.3
9/1/2009–8/31/2010	26	14	53.8
9/1/2010–8/31/2011	37	23	62.2
**Total**	**146**	**71**	**48.6**

### Sources of H5N1 Surveillance Data

Laboratory confirmed reports of HPAI H5N1 outbreaks in poultry and other birds were obtained from two sources: the online surveillance reports submitted by Egypt to the World Organisation for Animal Health (OIE) [11], and the EMPRESi database maintained by the UN Food and Agriculture Organization (FAO-list website source). These records contain information on the date, location, species involved, number of affected and culled animals. OIE data were used for 2006–2008, after which the EMPRESi data became more complete and were therefore used for subsequent years.

Data on human H5N1 cases in Egypt between 2006 and 2011 were obtained from the situation update archives on the WHO avian influenza website [3]. These represent laboratory confirmed cases reported by Egypt to WHO. For some of the human cases, the situation updates listed the apparent date of disease onset, while for others only a date of hospitalization or death was available. For the purposes of analysis, two of the authors independently reviewed the situation updates and recorded, for each human case, the earliest associated date. A process of consensus resolved any discrepancies between the dates recorded by the two observers. The location provided for most of the human cases in the WHO situation updates was at the governorate level; only rarely was more detailed geographic information available on human cases.

### Demographic Variables

Statistics for gross domestic product (GDP), human population density, and urbanization of each Egyptian governorate were obtained from United Nations Human Development reports [12] and demographic databases [13]. Poultry density maps for Egypt published by the UN Food and Agriculture Organization (FAO) were obtained from the FAO website [14]. Using ArcGIS 9.2, shape files of the Egyptian governorates [15] were used to analyze these poultry density maps and calculate a value of poultry density for each governorate. These statistics were included in the multivariate risk models.

### Climate Data

Daily records for temperature and humidity for each day of the study period (2005–2011) in the principal city of each governorate were downloaded from a publicly available website [16]. For governorates without weather data, we assigned the nearest governorate as a proxy. These data were used to calculate rolling 30-day average temperature and humidity values for each governorate during the study period.

### Temporal Comparison of Human and Bird Surveillance

We performed three types of temporal comparisons of human and bird H5N1 surveillance data: superimposition of the epidemiologic curves, a statistical permutation test of temporal concordance, and calculation of time lags between the first human and bird cases in a governorate. For these comparisons, the occurrence of a bird outbreak (rather than the total number of birds affected) was used, since the available data for outbreak size was noted to be incomplete and highly variable.

First, the epidemic curve for bird H5N1 outbreaks between 2006 and 2011 and the epidemic curve for human H5N1 cases over the same period were superimposed to allow for descriptive visual comparison.

Next, we tested for temporal concordance between the time series data sets of human H5N1 cases and bird H5N1 outbreaks using a permutation test that was constructed as follows. Data from the two time series were aggregated over calendar weeks. To smooth the noisiness of the surveillance data, the average of counts was calculated over a three-week period.

To test concordance of the human and bird H5N1 surveillance data streams, we performed a permutation test that calculated the sum of the squared difference between the normalized event values (number of human cases and number of bird outbreaks) for different temporal alignments of the data. The distribution of the set of all possible alignments was estimated. From this distribution, individual alignments could be statistically evaluated. To estimate the distribution of alignments, we began by letting the vectors *h* and *b* represent the values for human H5N1 cases and bird H5N1 outbreaks in a particular week. We defined the distance between these two vectors as the sum of the square of the individual elements, this also served as the test statistic *T*.
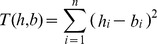



We defined *n* as the number of values in each of the time series and let *h_i_* and *b_i_* denote the *i*th elements of the human and bird time series respectively.

Next, we let *B* denote the circulant matrix generated by the vector *b*:
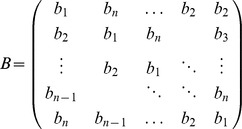



The columns of *B* correspond to rotations on the original vector *b*. The distance between the vector *h* and the *j*th rotation of B can then be calculated as:
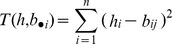
where *B_i,j_* is the element in the *i*th row and *j*th column of *B*. We postulated that if there was no temporal association between human H5N1 cases and bird H5N1 outbreaks then T(*h, B•j*) would not be significantly different than the distance between *h* and any of the other rotations of *b*. Using this as the null hypothesis, a p-value can then be calculated by finding T(*h, B•j*) for each 1<*j*< = *n* and then by finding the proportion of test statistics smaller than T(*h, B•j*), which is the test statistic for the alignment of the data. It should be noted that since, under the null, rotations are distributed uniformly at random, this is equivalent to sampling columns of *B*, finding the test statistic, and then finding the proportion of random alignment at least as small as T(*h, B•j*). Since the p-value of the data alignment was 0.01% (see Results), we were able to reject the null hypothesis.

Second, the time lag between reported bird and human cases was analyzed by determining the number of days between the date of the first animal case reported in a particular governorate and the first human case reported in the same area. Because the epidemic curve demonstrated on visual inspection that there was an apparent seasonal cycling of cases in both birds and humans, with highest rates during the winter season and lowest rates in the fall, we calculated time lags for each of the three September-September periods between 2005 and 2011.

### Multivariate Risk Modeling

We performed bivariate and multivariate analyses of a number of risk factors for prediction of human cases of highly pathogenic avian influenza H5N1in Egypt. The outcome measure was the rate of occurrence of WHO-confirmed human cases of HPAI H5N1 in a particular governorate, expressed as an incidence rate (cases per million persons). A Poisson regression model was used to analyze the association between the risk factors and the rate of human cases due to the rarity of the event and that the outcome variable was the count of human H5N1 cases on a particular day (on most days where cases occurred, there was only one case reported in the country). The ratio of the deviance to degrees of freedom (value/DF) was 1.038, indicating an acceptable goodness of fit of the data for the Poisson distribution. The independent variables analyzed included the demographic variables for each governorate described above. Other independent variables included the average temperature and humidity for the previous 30 days in each governorate. Finally, we analyzed as an independent variable the occurrence, in the previous 14 days or 21 days in the same governorate, outbreaks of H5N1 avian influenza in birds according to surveillance reports of OIE or EMPRESi records.

The multivariate model was constructed using independent variables that showed significant associations (p< = 0.1) with the outcome of human cases in the bivariate analysis. Next we tested for collinearity between independent variables. Any two independent variables with a Pearson's correlation coefficient greater than 0.6 were considered collinear. For example duck and chicken outbreaks were considered collinear, as were temperature and humidity. If this was the case, the collinear variable that provided the greatest explanatory value as determined by the Akaike Information Criterion (AIC) was allowed to remain in the multivariate model. In a similar fashion, we tested whether outbreaks in ducks or chickens conferred greater risk, and whether a time lag of 14 days or 21 days between a bird outbreak and a human case yielded the greatest predictive value for the final model (smallest value for AIC).

### Positive Predictive Value and Sensitivity

We calculated the positive predictive value for a bird outbreak by determining the proportion of bird outbreaks over the study period that were followed by a human case of H5N1 in the same governorate over the subsequent 2 week period:
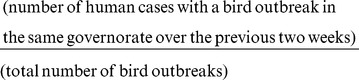



Similarly, we calculated the sensitivity of bird outbreaks to predict human cases as the proportion of human cases that were preceded by a reported bird outbreak in the previous two weeks, or:
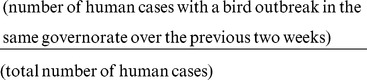



## Results

### Temporal and Spatial Patterns in Animal and Human Cases of H5N1

Figure 1 shows the epidemic curves for bird outbreaks and human cases of H5N1 HPAI in Egypt, by month, during the years 2006 through 2011. As Figure 1 shows, after an initial intense period of disease spread in 2006 following introduction of virus into the country, the pattern of bird and human cases appear to develop a cyclic pattern, with peaks in the winter months. In general, the number of bird outbreaks exceeds the number of human cases for most time periods.

### Temporal comparison of data streams at the national level

Figure 2 shows the aggregated, filtered curves for human cases and bird outbreaks of H5N1 in Egypt between 2006 and 2011, normalized to a similar scale. This statistical smoothing process (see Methods) highlights the cyclical nature of the disease events.

The degree of linkage or concordance between these two normalized curves was tested by a permutation test (see Methods) to see whether shifting one of the curves in either direction in time improved the statistical fit (the sum of the squared difference between the normalized event values) for human H5N1 cases and bird H5N1 outbreaks. It was found that the temporal alignment implied by the data was highly significant (p = 0.01) suggesting a temporal concordance between the bird and human cases. Among all possible alignments tested in the permutation test, it was found that the greatest concordance, that is the alignment that minimized the mean square error, was for a two week lead in human cases vs. bird outbreaks at the country level. In other words, an increase in human cases tended to appear prior to a corresponding increase in bird outbreaks during the annual cycles of infection.

### Spatial distribution by governorate of human cases and bird outbreaks

Figures 3A and 3B show the pattern of occurrence of bird outbreaks and human cases of H5N1 influenza A in the different Egyptian governorates over the time period. These figures show that the majority of bird outbreaks and human cases in Egypt have occurred in governorates located along the Nile River and in the delta region. These patterns reflect the higher human and poultry density that is present in governorates located near the Nile River, as shown in Figures 4A and 4B.

### Time Lags at the Governorate Level between First Report of Bird Outbreaks and Human Cases

Table 1 shows the actual time lags (number of days) between the date of the first bird outbreak from a particular governorate, and the date of the first reported human case in that governorate over 6 different yearly time periods between September 2005 and September 2011. In the first year and in general, reported bird outbreaks preceded reported human cases by time lags ranging as long as 294 days (average time lags each season ranging from 42–138 days) but this was not always the case. In several instances, the first human case in a particular season in a governorate preceded any reported bird case in the same administrative district. These instances appear as negative values for the time lags in the table.

### Predictors for Human H5N1 Case Occurrence at the Governorate Level

Table 2 shows the results of predictive modeling (using Poisson regression) of risk factors for the occurrence of a human case of H5N1 influenza A infection in a particular Egyptian governorate over the study period. A number of factors showed a significant bivariate association with the risk of a human case, including a lower temperature over the previous 30 days, lower percentage of urbanization of the governorate, lower GDP, higher poultry density, and the occurrence of a bird outbreak in the previous time period. Bird outbreaks in the previous 14 days were more strongly associated with human case risk than a case in the past 21 days. In the multivariate model, factors remaining significant included lower mean temperature in the preceding 30 days, lesser urbanization of the governorate, higher poultry density, and the report of a bird outbreak in the governorate involving either chickens or ducks during the preceding 14 days. This model had the best AIC value of the other possible models tested (AIC  = 1915.5).

The positive predictive value and sensitivity for a bird outbreak predicting the occurrence of a human case, is shown in Tables 3 and 4 for each of the annual disease cycles as well as the entire observation period. The positive predictive value, i.e. the probability that a bird outbreak will be followed by a human case in the same governorate during the subsequent 14 days, ranged from as low as 3% to more than 15%, with an average of approximately 12% (indicating a greater than 1 in 8 chance of seeing a human case in the governorate in the two weeks after a bird outbreak). The sensitivity indicates the proportion of human cases that were preceded by a reported bird outbreak in the previous two weeks. The sensitivity of bird outbreaks for subsequent human cases was high in the first year of the outbreak (85%), but fluctuated in later years, with an overall rate of almost 50%. This implies that 50% of the human cases occurred without a bird outbreak being reported in the governorate in the preceding 2 weeks.

## Discussion

Results of this study demonstrate both the potential value and challenges of comparing animal and human surveillance data streams for avian influenza H5N1 at a national and local level in a country such as Egypt that is experiencing ongoing outbreaks of infection in both humans and birds. Our analysis of these data streams between 2006 and 2011 indicates that there are linkages between the cyclical pattern of case occurrence in birds and humans. At the governorate level, the date of the first reported animal outbreak generally (but not always) preceded reported human cases during a given season. In a multivariate model, a poultry outbreak in the previous 14 days was a risk factor for the occurrence of a human case in a governorate. Other predictive factors included lower temperature in the previous 30-day period, lower urbanization and GDP of the governorate, and higher poultry density. The positive predictive value and the sensitivity of bird outbreaks to predict human cases in the same governorate within a two week period fluctuated over time, but in general were low.

The analysis had a number of important limitations. It relied on surveillance data of human and bird cases reported first to national agencies and then international agencies. While this suggests that the data were in compliance with the standards of those international organizations (the World Health Organization for human cases and the World Organisation for Animal Health/OIE and the UN Food and Agriculture Organization/FAO for bird cases), surveillance data are inherently subject to bias, and it is likely that the true incidence of both human and avian infection with HPAI differed somewhat from what was reported. Passive surveillance such as these data relies on recognition and reporting of clinically recognized cases. Subclinical cases or asymptomatic shedding of virus, which has been reported in vaccinated birds, would not be detected by passive surveillance. For some of the analytic comparisons of bird and human data, we used the count of a bird outbreak, rather than the number of birds affected. It should be noted that this aggregate outbreak measure will weight an outbreak over a small population, such as a rural farm, equal to an outbreak to a large breeding facility. While this choice of weighting is not ideal, individual bird outbreak data was found to be incomplete and variable and this aggregate approach helps mitigate underreporting in smaller production situations. It is also possible that subclinical cases in humans could go undetected by surveillance efforts; however, studies in other countries have rarely found significant evidence of subclinical human infection with H5N1 HPAI [17]. Other possible biases in the data could include enhanced surveillance for human cases in an area that is experiencing an intense epizootic, or, conversely, enhanced surveillance for poultry cases occurring after the diagnosis of a human case in the same region. Misclassification of data could occur in numerous ways, such as a human case seeking medical care in a different governorate where infection took place, leading to exposure misclassification.

Despite such limitations, these publicly available surveillance data represent an important assessment of human and avian infection in Egypt over this time period, and the value of integrating the human and animal data streams is supported by a number of biologically plausible results.

The statistically significant linkage between the human and animal occurrences of H5N1 HPAI indicates that there are shared environmental factors driving infection in both humans and animals [18]. Since bird outbreaks were more numerous than human cases, examining both data streams could make it easier to detect such patterns. The permutation test demonstrated that the two curves were closely linked in time. The fact that both the animal and human cases occurred in a cyclic pattern during the year with peaks in the late winter has been reported elsewhere [9]. The best fit of the permutation test showed that the human cycles preceded the bird cycles by approximately 14 days. This could be an artifact of the statistical smoothing process or a reflection of enhanced surveillance in human populations. The finding of peaks in the late winter months for both birds and humans could relate to our finding in the multivariate model that decreased temperature in the previous 30 days was associated with an increased risk of human H5N1 infection. This is also in agreement with experimental studies that have found that increased environmental temperature decreases the efficiency of influenza A transmission [19] and the survival of influenza on surfaces [20], [21], [22]. It is possible that climate factors affect environmental transmission of influenza through aerosols or surface contact even in a subtropical country such as Egypt. It is also possible that crowding of birds and contact between birds and humans could be greater during colder weather periods.

The utility of using bird outbreak information to predict human infection risk was supported by the finding that first outbreak reports in birds usually preceded reports of human cases at the governorate level, and that bird outbreaks, along with lower GDP and urbanization and higher poultry density, were a risk factor for the occurrence of a human case. The fact that a predictive model could be constructed using surveillance case reports and demographic data available at the governorate level suggests that such data could be used to provide ongoing mapping of human risk. This could supplement existing risk assessment efforts by identifying future hotspots that experts might not have suspected, and allow for enhanced preventive efforts. Alternatively, if the pattern of human cases deviates markedly from the predictions of the model, it could be an indication of altered transmission dynamics and routes of introduction.

At the same time, predictive models for zoonotic disease occurrence should receive the same evidence-based scrutiny as medical diagnostic tests in order to determine their practical usefulness. We assessed the positive predictive value and sensitivity of a bird outbreak in a governorate for predicting a human case within a two-week period. The results indicated that, on average, only one time out of eight did a human case follow a reported bird outbreak within 2 weeks in the same governorate. In addition, approximately 50% of the reported human cases occurred without a reported bird outbreak in the two preceding weeks in the same governorate. These sobering data are a reminder that zoonotic transmission of H5N1 HPAI is relatively rare, and the risk of human cases in Egypt remains low even in the presence of bird outbreak activity. At the same time, the yearly fluctuation in results suggests variability in disease control and reporting effectiveness.

Further extension of these integrated surveillance modeling approaches appears warranted. Future analyses could include viral strain and other genomic data from viruses isolated from human and animal cases, since some strains of avian influenza may more easily cross the species barrier. Developing more precise data sets for both human variables such as the location of the human cases (for this analysis only available at the governorate level) and greater accuracy of poultry density mapping and demographic data including the quality and extent of veterinary services could also help refine the risk analyses by using techniques such as spatial cluster analysis in a time-dependent fashion. Achieving these goals would necessarily involve continued quality assessment and improvement in animal and human disease surveillance capability and effectiveness for zoonotic influenza.

In addition to prediction of human risk, this study suggests other benefits of systematically comparing human and animal disease surveillance data streams for H5N1 influenza A. We found that the time lag between first reported bird outbreak and first reported human case varied widely between governorates. Reasons for this variation could include regional differences in the quality and completeness of surveillance systems and reporting. It is possible that in some areas, resources for surveillance and diagnosis of human cases exceeds that available for animal disease detection and reporting. In such cases, humans may serve as the “sentinels” for the presence of disease in nearby animals. Ongoing analysis of such time lags could highlight gaps in surveillance systems and assist with quality control efforts. Unusual time lags could indicate data accuracy problems including misclassification of the actual location or date of human or bird cases and lapses in surveillance efforts. In addition, reasons for regional variability in time lags could include local differences in the intensity and insecurity associated with zoonotic exposures. One such difference could be the degree of zoonotic risk faced by workers in larger poultry confinement operations compared to exposures in the village setting (which was associated with increased human risk in our analysis). Unusual time lags between reported bird outbreaks and human cases may also indicate a novel form of disease introduction, either through wild birds, poultry trade, environmental contamination, or a change in transmission patterns such as increased efficiency of human-to-human transmission. Overall, we believe that our findings argue for greater collaboration between animal and human health surveillance efforts for zoonotic influenza, and ongoing integration of case surveillance data streams with incorporation of climate and demographic data for enhanced prediction, as well as the possibility of coordinated outbreak investigation of both human and animal populations.
